# The Use of Metal Oxides (Al_2_O_3_ and ZrO_2_) and Supports (Glass and Kaolin) to Enhance DBD Plasma-Catalytic CO_2_ Conversion

**DOI:** 10.3390/ma18235411

**Published:** 2025-12-01

**Authors:** Agata Dorosz, Krzysztof Zaraska, Michał Lewak, Artur Małolepszy, Jakub Jaworski, Arkadiusz Moskal

**Affiliations:** 1 Faculty of Chemical and Process Engineering, Warsaw University of Technology, ul. Waryńskiego 1, 00-645 Warsaw, Polandartur.malolepszy@pw.edu.pl (A.M.); jakub.jaworski10.stud@pw.edu.pl (J.J.); 2 Łukasiewicz Research Network—Institute of Microelectronics and Photonics, Kraków Division, ul. Zabłocie 39, 30-701 Kraków, Poland; krzysztof.zaraska@imif.lukasiewicz.gov.pl

**Keywords:** plasma catalysis, dielectric barrier discharge reactor, CO_2_ conversion, low-temperature plasma, energy efficiency, packing materials/supports, metal oxides

## Abstract

Background: The conversion of carbon dioxide (CO_2_) into valuable products like carbon monoxide (CO) is an important process facing limitations due to poor energy efficiency. Dielectric barrier discharge (DBD) plasma reactors offer a potential solution through synergistic plasma catalysis, making the selection of an optimal solid packing material a critical design challenge. Methods: This study investigated the impact of four different packing materials—Al_2_O_3_, ZrO_2_, glass beads, and kaolin pellets—on the CO_2_ conversion process in a DBD reactor. The materials’ physical and chemical properties (porosity and composition) were analyzed. Experiments were conducted to examine the influence of gas flow rates and bead size on CO_2_ and CO concentrations. The study utilized optical emission spectroscopy (OES) and kinetic mathematical modeling to characterize the discharge and the reaction. Results: Higher gas flow rates led to a decrease in CO_2_ conversion due to reduced specific energy input. The addition of solid packing significantly improved system efficiency by promoting filamentary and surface discharges, with smaller beads yielding higher conversion rates. Notably, kaolin demonstrated unique performance characteristics, suggested by its increased plasma brightness, likely due to flow-induced turbulence promoting the reaction. Conclusions: Proper material selection and packing design are crucial for efficient CO_2_ splitting, concurrently boosting energy efficiency and maintaining high conversion. While Al_2_O_3_ (corundum) shows high intrinsic activity, kaolin emerges as a highly competitive and advantageous material when associated costs are considered paramount for large-scale applications.

## 1. Introduction

The escalating concentration of atmospheric carbon dioxide poses a significant global challenge, demanding the utilization of this waste and converting it into a new feedstock [1,2]. It has resulted in a booming interest in new technologies that adhere to the principles of sustainable and green chemistry, as well as help to achieve clean energy. The goal is not only to reduce CO_2_ (carbon dioxide) emission per se but also to effectively close the carbon loop by putting it back in use as value-added carbon sources following the cradle-to-cradle principle [3,4,5].

At present, sequestration and direct decomposition of CO_2_ selectively into carbon monoxide (CO) and oxygen (O_2_) attract attention from the scientific and industrial community. CO can serve as a reactant to produce higher-energy compounds. It can also be used for fuel synthesis and for the production of chemicals, such as esters and organic acids [6]. However, CO_2_ conversion is at a much lower technology readiness level and is hampered by several limitations, specifically poor energy efficiency, low product yield, and insufficient product selectivity [7]. There are still some hurdles to overcome because CO_2_ is a relatively thermodynamically stable molecule, and its splitting into CO and O_2_ is an endothermic process that is only favorable at very high temperatures [3,8]. Besides the requirement of substantial energy, the development of active catalysts that can break the bond in CO_2_ is imperative for the chemical conversion to take place. Thermal CO_2_ splitting with the use of catalysts has exhibited limited efficacy thus far. Beyond the traditional thermal route, several alternative CO_2_ conversion technologies are being actively investigated. Electrochemical reduction leverages electrical energy to produce valuable products (like CO or formic acid) under milder conditions, enabling integration with renewable sources. Similarly, photochemical approaches utilize specialized photocatalysts and solar irradiation for sustainable, low-energy conversion. Biological pathways, such as photosynthesis, are also key. While first-generation biofuels faced controversy due to competition with food agriculture, the use of microalgae offers a promising, economically viable route for biofuel production by efficiently capturing CO_2_ from various sources without high land or water demands. All these diverse methods—electrochemical, photochemical, and biochemical—contribute uniquely to CO_2_ utilization, with current research focused on optimizing their efficiency, scalability, and economic viability for sustainable material development [1,3,9,10,11].

Recently, the obstacles imposed by traditional thermal technology elucidate why some researchers have shifted their focus toward the combination of heterogeneous catalysis and plasma activation, i.e., plasma catalysis [12,13,14,15,16,17].

Non-thermal plasma (NTP) in the reactor is generated by applying a potential difference across parallel electrodes in a gas-filled apparatus. This creates an electric field that leads to the formation of positive ions and accelerated, highly energetic electrons [18]. The gas is activated through inelastic collisions with the electrons, rather than via bulk thermal heating. This unique characteristic facilitates the enhancement of chemical conversion and the occurrence of endergonic reactions, such as the splitting of CO_2_, by reducing the required energy expenditure [3].

One of the most common types of NTP reactors reported in the literature is the dielectric barrier discharge reactor (DBD) [3,16,19]. This apparatus is composed of two plane-parallel or concentric metal electrodes, and in the packed-bed configuration (DBD-PB), the electrodes are also separated by at least one dielectric barrier (e.g., glass, quartz, ceramic, or polymer materials) [20]. In DBD-PB reactors, catalysts are either embedded within the dielectric barrier as beads or coated onto its surface, so the structure is defined by two key components: a support and an active phase [21].

A key characteristic setting plasma-catalytic processes apart from conventional catalysis is their two-way, reciprocal interaction: alterations in the plasma state affect the catalyst, and the catalyst, in turn, impacts the plasma [7,22]. Plasma catalysis synergy includes changing the catalyst’s physicochemical properties, forming a hot spot, lowering the activation barrier, and changing surface reaction pathways. Shifting the focus, the active phase acts as a plasma catalysis promoter (PCP) rather than a real chemical catalyst, facilitating reactions not only at the catalyst surface but also within the plasma phase [3,7,21,23]. It induces alterations in plasma behavior by varying its intensity, contributing to electric field enhancements and, potentially, enhancing the number and intensity of micro- or surface discharges. For this reason, the ideal choice of catalyst in this case is not straightforward. In thermal catalysis, the mechanisms responsible for selective CO production are well understood. Catalyst addition primarily alters the intensity of adsorption and desorption processes and modifies the affinity between the converted gases and the bed material. Conversely, based on existing literature within the plasma-catalytic scope, the precise mechanism governing the analyzed process occurring in PB-DBD reactors remains unclear.

Various plasma-assisted catalytic strategies have already been explored to increase CO_2_ conversion and maximize energy efficiency. Currently, metal oxides constitute the predominant class of employed catalysts in reported studies [24,25]. Several materials have been investigated, including glass wool, glass beads, silica gel, quartz, quartz wool, quartz sand, Al_2_O_3_, CaTiO_3_, ZrO_2_, SiO_2_, BaTiO_2_, MgO, and CaO [26,27,28,29,30]. Michelsen et al. [27] demonstrated a process in a DBD-PB reactor filled with glass wool, quartz wool, and SiO_2_, ZrO_2_, Al_2_O_3_, and BaTiO_3_ spherical beads of different sizes, with BaTiO_3_ experiencing the highest conversion and energy efficiency. Yu et al. [26] investigated CO_2_ conversion in a packed-bed DBD reactor using silica gel, quartz, α-Al_2_O_3_, γ-Al_2_O_3_, and CaTiO_3_. Their findings demonstrated that introducing dielectric materials into the plasma reactor enhances the electric field, boosting electron energy and leading to higher CO_2_ conversion. Similarly, a series of Ca*_x_*Sr_(1−*x*)_TiO_3_ materials has been explored for CO_2_ splitting in a DBD reactor [31,32,33].

This study investigates the pivotal role of packed-bed materials in enhancing the efficiency of CO_2_ conversion within a non-thermal plasma environment. The study utilized a coaxial cylinder dielectric barrier discharge reactor packed with a plasma-catalytic promoter to decompose CO_2_ into its constituent products, CO and O_2_. The experiments involved an argon/CO_2_ gas mixture and were conducted at different gas flow rates. The process occurred under atmospheric pressure and operating bed temperature (heated due to plasma). We conducted a systematic evaluation of a diverse range of catalytic materials and supports, encompassing Al_2_O_3_ (embedded on glass beads), ZrO_2_ (embedded on glass beads), and, for reference, pure glass beads (of 3 mm, 5 mm, and 10 mm in diameter) and pure kaolin pellets. Our study employed a selection of packing materials to cover both established and emerging candidates. Al_2_O_3_ serves as a baseline due to its prevalence in DBD applications, and ZrO_2_ allows for the investigation of intrinsic catalytic effects. Kaolin was specifically included as an inexpensive ceramic with high potential as a pellet material. Crucially, kaolin’s ease of surface functionalization—by introducing active species like CeO_2_ or Ni_2_O_3_—makes it particularly relevant for future optimization of plasma-catalytic synergy. Each material’s performance was rigorously assessed against key metrics: CO_2_ conversion and energy efficiency. Furthermore, a mathematical kinetic model was developed to provide insight into plasma dynamics. This model accounts for the chemical reactions taking place within the reactor under investigation, using a packed bed of corundum beads as an illustrative case study. The results of the research are intended to shed light on the rational design and the refinement of advanced plasma-driven catalytic systems for sustainable CO_2_ utilization. Notably, this study represents the first use of plasma chemistry spectroscopic measurements in a DBD reactor to investigate the influence of the bed material on the resulting products.

## 2. Materials and Methods

### 2.1. Materials

The glass beads of 10 mm in diameter were sourced from Yiwushiijiruishangamao Co., Ltd. (Yiwu, China).

The glass beads of other sizes (3 and 5 mm in diameter) were procured from BIOSPACE (Poznań, Poland).

The 3 mm corundum beads and the 3 mm zirconia beads were purchased from a producer in Wuping (Shenzhen, China).

The kaolin (clay) powder (Nr CAS: 1332-58-7) was supplied by Biomus sp. z o.o. (Lublin, Poland).

Carbon dioxide of technical grade (99.5% purity, Product Code: 252158) was supplied by Air Products and Chemicals Inc. (Trexlertown, PA, USA).

Argon of Premier Grade 5.2 purity (99.9992%, Product Code: 168046) was supplied by Air Products and Chemicals Inc. (Trexlertown, PA, USA), with impurity specifications as follows: THC < 0.1 ppm, H_2_ < 2 ppm, O_2_ < 1.5 ppm, and N_2_ < 4 ppm.

### 2.2. Methods

#### 2.2.1. Characteristics of Non-Thermal Plasma Packed-Bed Reactor with Dielectric Barrier Discharge

**Figure 1:**
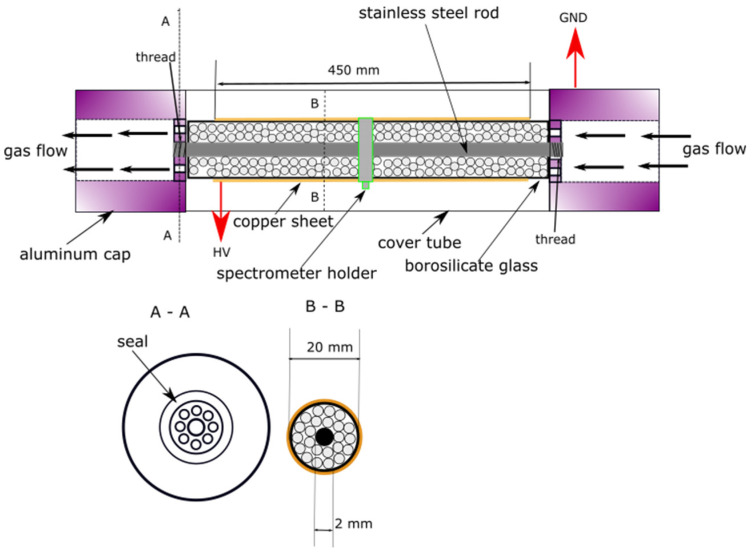
The schematic diagram of the DBD-PB reactor used in this study. (A-A) cross section of the holding head of the reactor, (B-B) cross section of the main part of the reactor.

Figure 1 depicts a schematic diagram of the reactor used in this study.

The reactor (DBD-PB configuration) consisted of a cylindrical, coaxial, borosilicate glass tube (D_in_ = 30 mm, L = 500 mm). A 2 mm steel rod serving as the cylindrical inner collecting electrode (grounded) and a copper foil acting as the outer discharge electrode (connected to a high-voltage output) on the outside surface of the glass reactor tube. The electrodes were separated by glass and a dielectric barrier packing. The reactor’s inlet and outlet sections, each 25 mm in length and free of electrodes, were consistently filled with 5 mm diameter glass beads. This packing prevented gas flow blockage at the inlet and outlet ports. The central section of the reactor, however, was filled with the packings analyzed in subsequent experimental variants. The volume of the empty reactor was equal to 2.83 × 10^−4^ m^3^.

Four packing materials (glass, corundum, zirconia, and kaolin) were tested to compare the differences in plasma generation in the DBD-PB reactor. Two different pellet shapes (sphere and cylinder) and a series of spherical pellet sizes (3, 5, and 10 mm in diameter) were explored for CO_2_ splitting in the reactor. The studied packing variants were as follows: glass beads (3, 5, and 10 mm, respectively), corundum beads (3 mm), zirconia beads (3 mm), and cylindrical kaolin pellets (10 mm in diameter, with a 1:1 diameter-to-height ratio). The masses of the reactor packings for each variant were as follows: 0.333 g for 3 mm glass, 0.303 g for 5 mm glass, 0.257 g for 10 mm glass, 0.276 g for corundum, 0.421 g for zirconia, and 0.122 g for kaolin. Table 1 presents the summary of the geometric parameters of the packing beds along with the masses of the single bead/pellet and the whole packing bed.

#### 2.2.2. Production of Kaolin Pellets

One of the objectives of our experiments was to produce cylindrical kaolin pellets with a 1:1 diameter-to-height ratio, each measuring 10 mm. A slurry was prepared by dispersing 165 g of kaolin powder in 100 mL of water, used as a binding agent, to a desired consistency. To ensure compactness and dimensional homogeneity, the resulting clay-based mixtures were fully transferred to their designated molds and pressed to a consistent level. The as-fabricated green body specimen was initially air-dried for 24 h in a laboratory environment at a temperature of 25 °C. This was followed by demolding and a thermal treatment in an electric oven at 80 °C for 12 h. This secondary drying step was performed to remove residual moisture and to enhance the mechanical stability of the green body for subsequent handling.

After drying, the green bodies underwent thermal treatment by heating to the sintering temperature of 1000 °C in a muffle furnace. The procedure was carried out in the following manner: The specimen was heated at a constant rate of 4.5 °C/min until the target sintering temperature was reached. The temperature was then held for 2 h to produce the ceramic pellets. After that, the furnace was switched off for cooling, and the samples were taken after the furnace temperature had reached below 100 °C.

#### 2.2.3. Surface Area and Porosity Characterization of the Kaolin Pellets

The porosity of the ceramic pellets was characterized by determining their pore volume and specific surface area with a 3Flex porosimeter (Micromeritics, Norcross, GA, USA). The kaolin material was subjected to Brunauer–Emmett–Teller (BET) analysis specifically to verify its non-porous nature at the microscale, confirming consistency with the other reactor packing materials used. Details of the measurement procedure can be found elsewhere [34].

#### 2.2.4. Chemical Composition Determination of the Packing Beads

The chemical composition of the packing bead samples was determined using energy-dispersive X-ray fluorescence (ED-XRF) with an Epsilon 3XLE spectrometer (Malvern Panalytical; Almelo, The Netherlands). Qualitative analysis of the spectra was performed with the OMNIC ver. 1.4.G software package. Prior to measurement, the solid samples were placed in polypropylene (PP) holders sealed with a 3.6 µm Mylar film, with a diameter of 28 mm, prior to measurement. All measurements were performed using the built-in rhodium (Rh) X-ray tube of the instrument; therefore, the contribution from rhodium was excluded from the final composition calculations.

#### 2.2.5. Experimental Setup and Procedure

**Figure 2:**
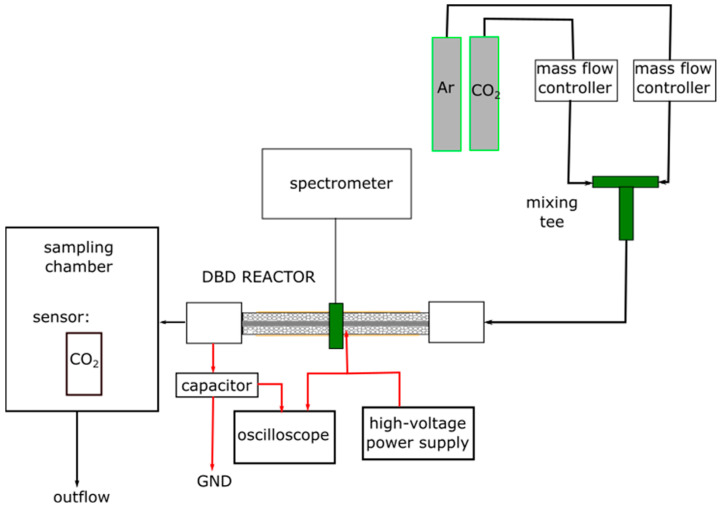
The schematic representation of the complete experimental setup. Red arrows marks the electric connections.

The schematic of the experimental system is shown in Figure 2.

The carbon dioxide was diluted in argon to reach a target CO_2_ concentration of 2500 ppm. Mass flow controllers were used to precisely regulate the flow rates of all gases. Specifically, CO_2_ flow was controlled by a model SFC6000D-50slm (Sensirion AG, Stäfa, Switzerland), and argon flow was controlled by a model GCR-C9KA-BA30 (Vögtlin Instruments GmbH, Muttenz, Switzerland). A series of experiments was conducted at argon flow rates of 3, 5, and 7 dm^3^/min. The dilution ratio remained constant for all experiments. The gas streams were introduced into a tee-connector for subsequent mixing. The resulting gas mixture was then directed to the DBD-PB reactor, operating at atmospheric pressure. After the DBD-PB reactor, the outlet gas was sent to a sampling chamber, and the concentrations of CO_2_ and CO were assessed by the measuring system for subsequent analysis [35]. It consisted of a CO_2_ sensor (SparkFun SCD41 Qwii SEN-22396, Niwot, CO, USA) and a CO sensor (series SEN04xx, DFRobot, Shanghai, China). These sensors provided rapid and straightforward measurements, which were validated using gas chromatography (GC-2014, Shimadzu, Kyoto, Japan).

The plasma was ignited by a high-voltage AC power supply (an alternating voltage with an amplitude of 9.5 kV and a frequency of 5130 Hz).

The experiment began after the reactor achieved thermal stability, reached by pre-heating the system with a pure gas flow for 20 min (argon, 3 dm^3^/min). To measure the temperature of the packing, a centrally placed corundum tube was used to house a thermocouple, which was subsequently immersed within the reactor bed. The conversion process resulted in the bed temperature stabilizing within the range of 70 to 100 °C (depending on the studied variant).

#### 2.2.6. Replicate Measurement Protocol

In summary, six packing variants were investigated, each for three different gas flow rates through the reactor, resulting in a total of 18 experimental conditions. For each experimental condition, the measurement was repeated nine times to obtain mean values and values of standard deviation.

#### 2.2.7. Electrical Characterization of the Discharge

The electric power applied to our experimental setup was measured in situ with a power meter and was equal to 195 W. The electrical parameters of the discharge were recorded using an oscilloscope. The applied voltage and the electrical current were monitored in situ. The measuring system enabled the temporary plasma power to be captured so as to calculate the time-averaged plasma power, P_av_, which was described in detail elsewhere [18,35].

#### 2.2.8. Imaging of Discharge Propagation

Images of the operating reactor were captured using an iPhone 14 digital camera (Apple Inc., Cupertino, CA, USA) camera to visualize the interaction between plasma and packing material and to provide a qualitative comparison of the modes of discharge propagation.

#### 2.2.9. Spectroscopic Analysis

**Figure 3:**
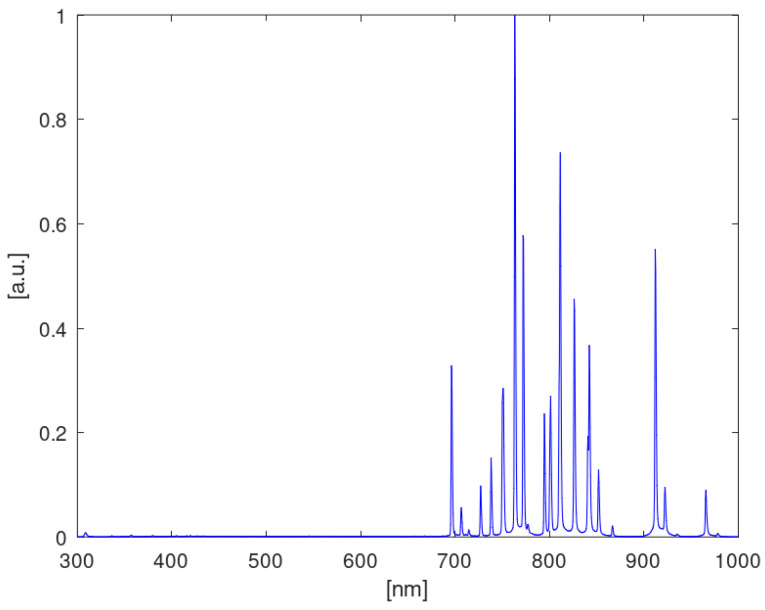
The optical emission spectrum of argon.

Plasma optical emission spectroscopy was performed using a spectrometer operating in the spectral range of 210–1080 nm (10083CAH apparatus, Hamamatsu Photonics, Hamamatsu, Japan). Preprocessing of spectral data was performed using SpectraGryph version 1.2.16.1 software (Dr. Friedrich Menges, Oberstdorf, Germany). Qualitative analysis was performed utilizing custom scripts in the GNU environment. The measurement methodology was as follows: For each bed material, researchers first acquired a dark spectrum (high-voltage power supply off) and then acquired a reference spectrum of pure Ar plasma at 3 dm^3^/min flow. Then, spectra were acquired operating on an Ar/CO_2_ mixture (2500 ppm CO_2_ in Ar) at flows of 3, 5, and 7 dm^3^/min. The dark spectrum was subtracted from both reference (Ar) and measurement (Ar/CO_2_) spectra before further processing. All observed plasma emissions were dominated by argon lines in the 700–1000 nm range (Figure 3). All obtained spectra were normalized using the maximum value corresponding to the 763.5 nm Ar* line.

Calculation of electron temperature has been performed on Ar I lines using the Boltzmann method following the description in [36]. Because the used spectrometer has a lower spectral resolution than the spectrometer used in [36], an alternate set of Ar lines was selected. The lines were chosen as a subset of lines used for the same purpose in [37] and characterized by a good signal-to-noise ratio in the obtained measurements.

#### 2.2.10. Conversion and Energy Efficiency Calculations

Two distinct types of conversion are considered. The first, referred to as the **absolute conversion**, is a widely used measure in the literature and is based on the molar flow rates of a specific reactant (e.g., CO_2_) [3]. This is the standard definition of “conversion” used throughout this review (expressed as a dimensionless fraction), calculated as follows:

(1) $$X_{{ABS,CO}_{2}}=\frac{{FCO}_{2},inlet-{FCO}_{2},outlet}{{FCO}_{2},inlet},$$

where *F*CO_2_,inlet stands for the molar flow rate of CO_2_ (in mol/s).

An alternative definition of conversion is also important, as it allows for a direct comparison of a reactant’s conversion rate across a gas mixture (e.g., with argon), highlighting how its intrinsic conversion is affected, rather than just the total amount converted. Taking dilution into account, the **effective conversion** [3], expressed as a dimensionless fraction, is given by the following formula:

(2) $$X_{{EFF,CO}_{2}}=X_{{ABS,CO}_{2}}\cdot \frac{{FCO}_{2},inlet}{{FCO}_{2},inlet+FAr,inlet},$$

where *F*Ar,inlet stands for the molar flow rate of argon (in mol/s).

The ratio of the plasma power to the total gas flow rate, known as the **specific energy input (SEI)**, is a crucial factor that governs both the conversion and energy efficiency in plasma systems [3]. Quantified in kJ/mol, it is defined by the following equation:

(3) $$SEI=\frac{P_{AV}}{{FCO}_{2},inlet+FAr,inlet},$$

where *P*_AV_ denotes the discharge (plasma) power obtained by the area calculation of the charge–voltage Lissajous figure (in kW).

The performance of a plasma process is also commonly evaluated by its **energy efficiency** [3]. Energy efficiency quantifies how effectively the process utilizes energy in comparison to the standard reaction enthalpy, with its value directly linked to the specific energy input (SEI):

(4) $$\eta_{E}=X_{{EFF,CO}_{2}}\cdot \frac{{∆H}_{298K}^{o}}{SEI},$$

where is ${∆H}_{298K}^{o}$, the enthalpy for pure CO_2_ splitting, and is equal to 283 kJ/mol.

**Energy cost** refers to the process energy consumption, typically expressed in kJ per mol of converted gas [3]:

(5) $$EC=\frac{SEI}{X_{{EFF,CO}_{2}}},$$

To accurately assess the reactor’s conversion efficiency from an economic perspective, a new performance metric was introduced. **Mass-normalized conversion efficiency factor per energy unit (*Ψ*)** is a parameter that quantifies the number of moles of CO_2_ converted per kilogram of packing (*m*_BED_) for every kilojoule of supplied energy:

(6) $$\Psi =\frac{{FCO}_{2},inlet\cdot X_{{ABS,CO}_{2}}}{P_{AV}\cdot m_{BED}},$$

This metric allows for a comprehensive evaluation of different operating variants, with higher values signifying increased process efficiency.

## 3. Results and Discussion

### 3.1. Specific Surface Area and Porosity Characteristics of the Kaolin Pellets

The BET specific surface area of the kaolin sample was determined to be equal to 11.247 m^2^/g. The value of BJH cumulative pore volume was determined to be 8.95 cm^3^/g (adsorption) and 10.163 cm^3^/g (desorption). The value of the BJH average pore diameter was found to be 22.988 nm (adsorption) and 20.294 nm (desorption).

The physicochemical properties of the packing, particularly its pore structure, are crucial for synergistic effects in packed-bed DBD reactors. While a high specific surface area is a key factor in conventional catalysis, our findings suggest that a high pore volume and macroporous structure play a dominant role in this system. It is important to note that this high pore volume primarily represents the inter-particle void space, which is crucial for overall gas flow and plasma penetration. This suggests the observed high energy efficiency is primarily due to the careful tuning of plasma discharge conditions rather than traditional heterogeneous catalytic effects. This point will be discussed in greater detail in the following sections of this article. The structure of kaolin brick used as a bed in the reactor shows that it may be treated as a non-porous material at the nanoscale level, similar to other materials used. Despite this, the operative site for plasma chemistry is the inter-pellet void space (macroporosity). This plasma chemistry involves the generation and reaction of plasma-generated excited species and radicals. Therefore, the overall bed macroporosity, as quantified in Table 1, serves as the critical parameter. Moreover, of note is the fact that kaolin’s suitability as a meso- and microporous catalyst support is attributed to its abundance and low cost. Its layered structure, combined with exceptional thermal and mechanical stability, makes it a highly versatile material for the fabrication of composites and hybrids [38]. Collectively, these attributes establish kaolin as a promising candidate for use as a plasma promoter in the analyzed packed-bed DBD reactors.

### 3.2. Chemical Composition Analysis of the Packing Beads

ED-XRF analysis revealed the chemical composition of the samples, with the detailed elemental breakdown compiled in Table 2 and Table 3. The data includes the elements detected in the analyzed samples, expressed as percentage content without normalization to 100%.

Based on Table 2, it can be concluded that the glass supports of 3 mm and 5 mm exhibit the same composition regardless of diameter. Differences in the percentage content of elements result from the irregular shapes of the samples. The larger the diameter, the smaller the surface area from which the signal is collected (since the material was not powdered). Spheres with a diameter of 10 mm differ in the content of individual components.

In the case of the other supports shown in Table 3, the zirconia support was produced from zirconium oxide stabilized with yttrium oxide, with the addition of zirconium silicate and alumina. The corundum spheres, on the other hand, contain significant amounts of silicates. The composition of the kaolin support is like that of the corundum support, where the Si/Al ratio is approximately 1/2 and 1/3, respectively.

### 3.3. Conversion and Energy Efficiency Analysis

**Figure 4:**
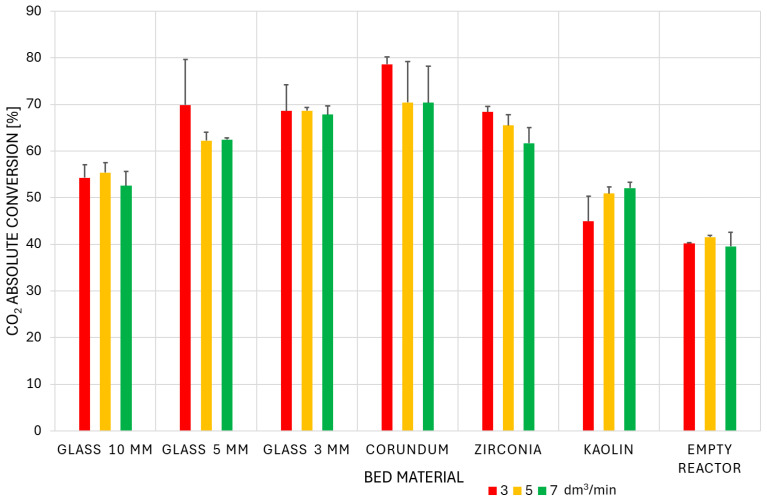
The absolute conversion of CO_2_ for all studied variants.

The comprehensive data on absolute conversion of CO_2_ (expressed in %) under various conditions were collected and are presented in Figure 4. These results represent the mean values and their standard deviations, calculated from repeated runs for each experimental condition.

Figure 4 shows that the conversion efficiency of the packed-bed reactor in the ascending order is as follows: empty reactor < kaolin < 10 mm glass < 5 mm glass < zirconia < 3 mm glass < corundum. Increasing the gas flow rate decreases the SEI, resulting in lower CO_2_ conversion because less energy is supplied per mole of reactant to drive the reaction. Furthermore, a higher gas flow rate results in a shorter residence time in the reactor, ultimately reducing the number of molecular collisions with energetic electrons and, consequently, the rate of bond destruction [39]. However, this also results in lower concentrations of CO and O_2_, making the reverse reaction less probable. Overall, high flow rates are preferred for improving reactor throughput and reaction rate. A notable exception to the aforementioned conversion–flow rate dependence was kaolin. This material did not follow the observed behavior. For kaolin, the character of the plasma discharge in the inter-particle void spaces may be a crucial factor. This is attributed to the influence of increasing flow turbulence, which in turn promotes the carbon dioxide conversion process. Additionally, for the empty reactor, the gas flow rate had no significant effect on conversion. In the case of variants with beads and pellets, the positive contribution of the packing compensates for the lower residence time, resulting in higher conversion compared to the empty reactor. The obtained results demonstrated good consistency across repeated runs.

Depending on the DBD packing and the flow of the gas mixture, the mean power consumed by the system (transferred for barrier discharge) at maximum voltage ranged from 55 W to 70 W (corresponding to the 28–36% power efficiency, as all systems consumed 195 W). The SEI parameter for each experimental condition was determined from the plasma power and the molar flow rates of CO_2_ and argon. The calculated SEI values are summarized in Table 4.

The maximum specific energy input of 31.6 kJ/mol was achieved with the corundum packing at an argon flow rate of 3 dm^3^/min. The results showed good repeatability.

**Figure 5:**
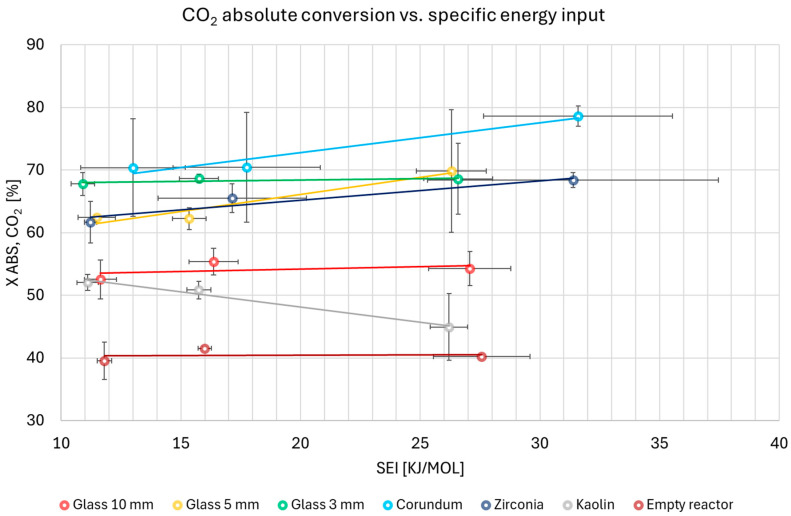
Absolute conversion of CO_2_ as a function of specific energy input for all studied variants.

Figure 5 displays the dependence of the absolute conversion of CO_2_ on the specific energy input. The reported results are mean values, along with the standard deviations across repeated runs, obtained for the respective experimental conditions.

The results demonstrated good consistency across repeated runs. The CO_2_ conversion efficiency increased sequentially with the following packing materials: empty reactor, kaolin, 10 mm glass, 5 mm glass (similar to zirconia), 3 mm glass, and corundum. This trend was consistent for specific energy input (SEI) values below 22.5 kJ/mol. Above this threshold, the conversion differences between the 5 mm glass, 3 mm glass, and zirconia packings were negligible. Based on the results obtained with glass beads of varying sizes, it can be concluded that particle size is a critical parameter influencing conversion efficiency [6,17]. Smaller packing beads generally lead to higher conversion by increasing the discharge surface area, raising breakdown voltage, and reinforcing surface discharges. However, this effect is highly dependent on the gas composition and can be mitigated or even reversed in the absence of a carrier gas like argon [29].

As the SEI increased (corresponding to a higher energy input per mole of CO_2_), the conversion behavior varied depending on the packing material:

- For 10 mm glass, 5 mm glass, zirconia, and corundum, conversion efficiency increased. This result is consistent with the expected outcome of supplying more energy to the process.
- For the empty reactor and 3 mm glass, conversion efficiency remained constant with increasing SEI. A reasonable explanation for the constant conversion efficiency is that a mass transfer limitation becomes the dominant factor. While the energy input increases, the conversion plateaus because the rate of CO_2_ molecules reaching the plasma-activated sites does not increase proportionally.
- For kaolin, conversion efficiency decreased with increasing the value of the SEI parameter. This may also be due to a different discharge character in the inter-particle spaces, which could be influenced by the higher flow rate.

**Figure 6:**
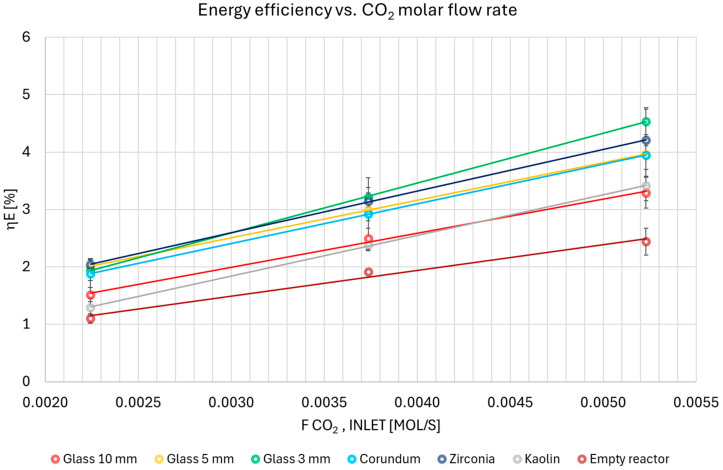
The energy efficiency at different inlet molar flow rates of CO_2_ and using different packings.

Figure 6 highlights how energy efficiency, expressed in [%], varies with the molar flow rate of CO_2_. The mean values for each test condition, together with their standard deviations, were computed based on the multiple runs performed. The limited magnitude of the standard deviation highlights the consistency of the results.

The experimental results, as collated in Figure 6, demonstrate a clear tendency. Energy efficiency consistently increased with a higher molar flow rate of CO_2_. The energy efficiency of the materials increased sequentially, starting with the empty reactor and ending with zirconia. The full hierarchy was as follows: empty reactor, kaolin, 10 mm glass, corundum, 5 mm and 3 mm glass, and zirconia. As the molar flow rate of CO_2_ increased, the ranking shifted. Kaolin’s efficiency surpassed that of 10 mm glass. Corundum and 5 mm glass started to exhibit comparable efficiency, while zirconia maintained its position as the most effective material. The 3 mm glass packing ultimately emerged as the most efficient variant. A key reason this analysis is so crucial is that it highlights the delicate balance between two primary objectives: achieving a high degree of conversion and ensuring high energy efficiency.

**Figure 7:**
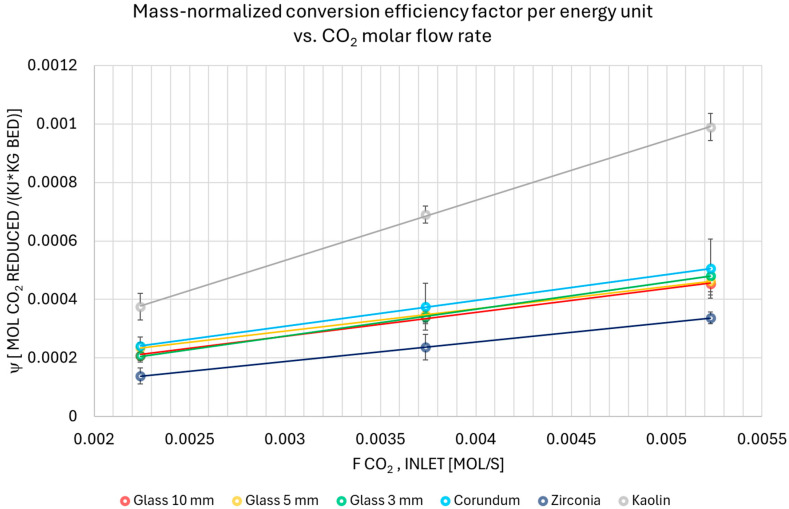
The mass-normalized conversion efficiency factor per energy unit at different inlet molar flow rates of CO_2_ and using different packings.

Figure 7 illustrates the impact of the molar CO_2_ flow rate on the mass-normalized conversion efficiency factor (*Ψ*), providing a clear comparison of the performance of different packing materials. For every experimental condition, the data points are shown as the mean value with the standard deviation derived from the repeated measurements. The data exhibited low variability (or low scatter), suggesting reliable measurements.

Figure 7 illustrates a consistent increase in the *Ψ* factor, progressing from zirconia and glass variants to corundum and kaolin. This trend is directly linked to the variability in the mass of the packing material across the tested set of packings, given the fixed bed volume. A lower packing mass facilitates a higher molar flow rate of CO_2_ and thus enhances process throughput.

These results highlight that kaolin is exceptionally favorable from an economic standpoint. As a material that achieves the highest conversion efficiency per energy unit, it simultaneously requires the lowest mass of packing. Consequently, kaolin not only allows for the strategic management of energy expenditure in the CO_2_ conversion process but also significantly lowers material costs, which is crucial for the scalability and sustainability of the technology.

For a definitive evaluation of the possible trade-offs in selecting a suitable reactor packing, Table 5 synthesizes the entire preceding discussion. This table provides a direct comparison of the packings based on conversion efficiency, energy efficiency, and cost, alongside the corresponding values of the novel qualitative parameter (mass-normalized conversion efficiency factor per energy unit).

Review studies report the difficulty in achieving high conversion efficiency concurrently with high energy efficiency (and associated low energy costs). In our view, from a material perspective, the mass of the reactor packing utilized in the process deserves equal consideration, as this factor significantly contributes to the costs that require minimization. Our objective was to propose a dimensionless factor whose maximization serves as an indicator of achieving the desired overall performance (i.e., maximizing conversion efficiency, maximizing energy efficiency, and minimizing the costs resulting from energy requirements and the necessary mass of the packing).

The favored materials for the analyzed process are 3 mm glass and corundum. Corundum exhibited the highest conversion efficiency and a relatively high energy efficiency and low energy cost, while the 3 mm glass beads yielded the highest energy efficiency and the lowest energy cost, along with a relatively high conversion at an argon flow rate of 3 dm^3^/min; however, increasing the flow rate was observed to deteriorate the obtained effects.

A distinguished alternative among all packings is kaolin because the process dependence on the inlet gas flow rate for this material shows an inverse trend. Increasing the flow rate of the gas mixture improves the absolute CO_2_ conversion, and its lower value (relative to 3 mm glass and corundum) is balanced by the significantly better reactor throughput (or processing capacity) under these operating conditions. Satisfactory values for energy efficiency and energy cost are also achieved. From an energetic and, most importantly, material perspective, the decisive parameter is the mass-normalized conversion efficiency factor per energy unit, as the variant using kaolin at the highest gas flow rate resulted in the most favorable conditions for the CO_2_ to CO conversion process.

### 3.4. Spectroscopic Analysis of Plasma Species and Electron Temperature

Optical emission spectroscopy (OES) was performed to identify the key reactive species generated within the plasma, providing insight into the discharge chemistry in the presence of various packing materials, and to measure electron temperature.

For electron temperature determination using the Boltzmann method, we follow the approach shown in [36,37].

**Figure 8:**
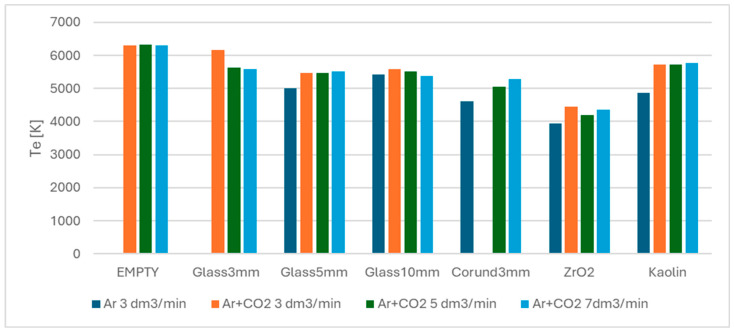
The comparative analysis of electron temperature across all studied variants.

The selected set of transitions is shown in Table 6. Calculated values of electron temperature are given in Table 7 and Figure 8. The obtained values are reasonably consistent, falling between approximately 3950 and 5750 K, with the median value of about 5050 K. In Table 6, “no data” denotes that the corresponding emission spectrum has not been captured due to an experimental error; thus, the electron temperature could not be calculated. Data series for zirconia exhibit a lower electron temperature. In this case, the lower electron temperature can be caused by a relatively high (>20) dielectric constant of the bed material, resulting in more power being expended on charging and discharging increased reactor capacitance.

**Figure 9:**
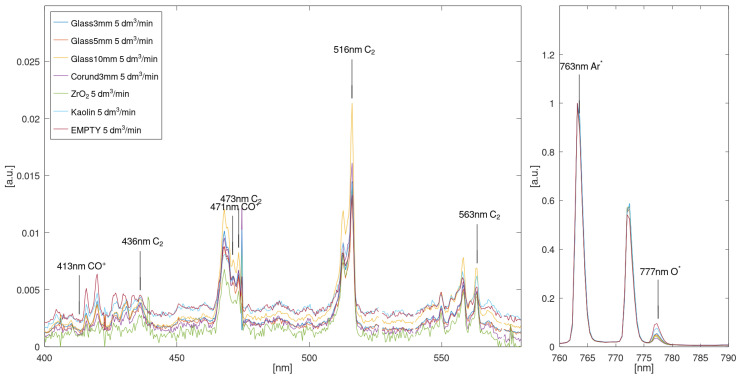
Spectra obtained for 5 dm^3^/min Ar+CO_2_ flow rate with different bed packing materials. Left panel: 400–580 nm range, showing characteristic peaks for studied CO^+^ and C_2_ emission lines. Right panel: 760–790 nm range, showing the 763 nm Ar* line used as a normalization target and the 777 nm O* line. Note the difference in vertical scale between the left and right panels.

Identification of active species, i.e., CO^+^, C_2_, O* and O_2_^+^ was performed using a subset of associated spectral lines proposed in [40]. The chosen subset of spectral lines is shown in Table 8. Spectra obtained at 5 dm^3^/min Ar+CO_2_ flow rate for all tested catalysts (after subtracting dark detector current) are shown in Figure 9.

**Figure 10:**
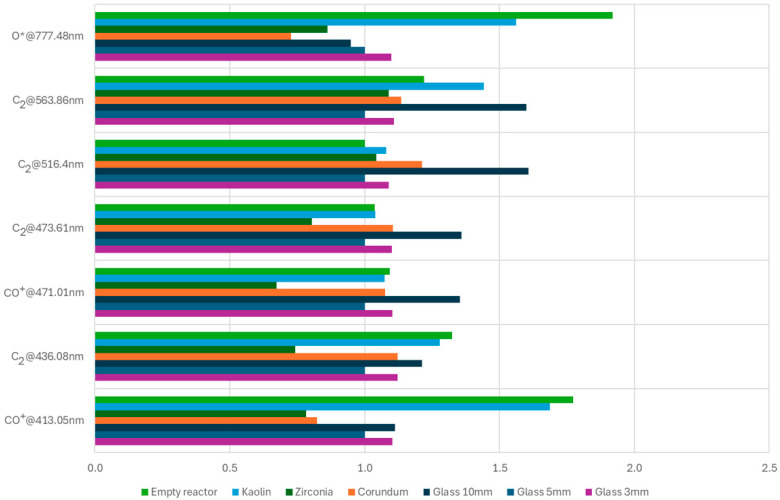
The obtained intensity values for different species for different bed materials normalized to glass bed.

Comparison of intensity values for different species shown in Figure 9 across bed materials is shown in Table 9 and Figure 10. Table 7 and Figure 10 contain brightness values for each spectral line, relative to the brightness of each line measured for a 5 mm glass bed (assumed to be 1). This presentation allows for easy determination if, for a given bed material, a given species is more (values above 1) or less (values below 1) active than it is in a reactor with a 5 mm glass bed. We note that the kaolin bed is characterized by increased plasma brightness for all species (CO^+^, C_2_ and O*), suggesting increased reaction efficiency compared to the reference glass bed.

The primary product of CO_2_ conversion is CO. During the process conducted in a low-temperature plasma, various intermediate species such as CO^+^, C_2_, and O* are generated, which can be confirmed through spectroscopic plasma analysis. The intensity of the CO^+^ emission line is proportional to the concentration of CO, while the intensity of the O* line correlates with the oxygen concentration produced during the process. The oxygen generated may oxidize CO to CO_2_ (an undesirable side reaction) or evolve into O_3_. The C_2_ emission line indicates the potential formation of higher carbon chain species.

An alternate approach to normalization of line intensity is shown in Table 10 and Table 11. Here, we present line intensities normalized to the intensity of the 777.48 nm O* line for CO^+^ and C_2_ systems, the median value for each system, and the catalyst ranking. We assume that the higher relative intensity of the C_2_ or CO^+^ system versus the O^*^ line corresponds to higher production of C_2_ or CO versus atomic oxygen. It can be seen from the data that the highest relative production of both CO and C_2_ happens with the corundum catalyst, while the lowest relative production happens with the empty reactor. We believe that due to the higher electron temperature and higher O* concentration in an empty reactor, the produced O* oxides CO and C_2_ back to CO_2_.

The obtained results suggest that the most suitable reactor filling material for CO production with C_2_ (and potentially higher hydrocarbons) is corundum (Al_2_O_3_). In contrast, for pure CO production without C_2_ formation, zirconia (ZrO_2_) provides the best results. If the reactor is intended for oxygen production from CO_2_, such as in the context of oxygen generation from the Martian atmosphere, an unfilled reactor configuration appears to be the most effective.

### 3.5. Discharge Visualization and Analysis

**Figure 11:**
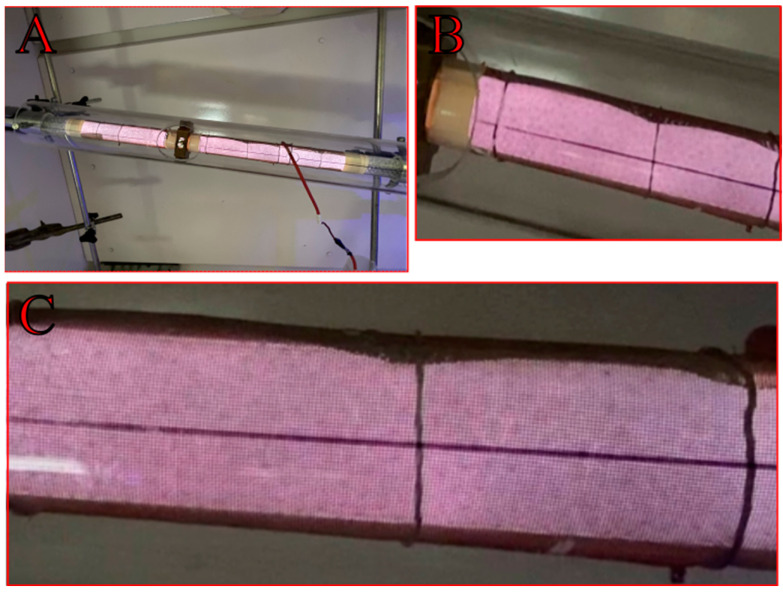
Exemplary imaging of plasma discharge in the DBD reactor packed with 3 mm glass beads (**A**); ((**B**,**C**) zoom).

**Figure 12:**
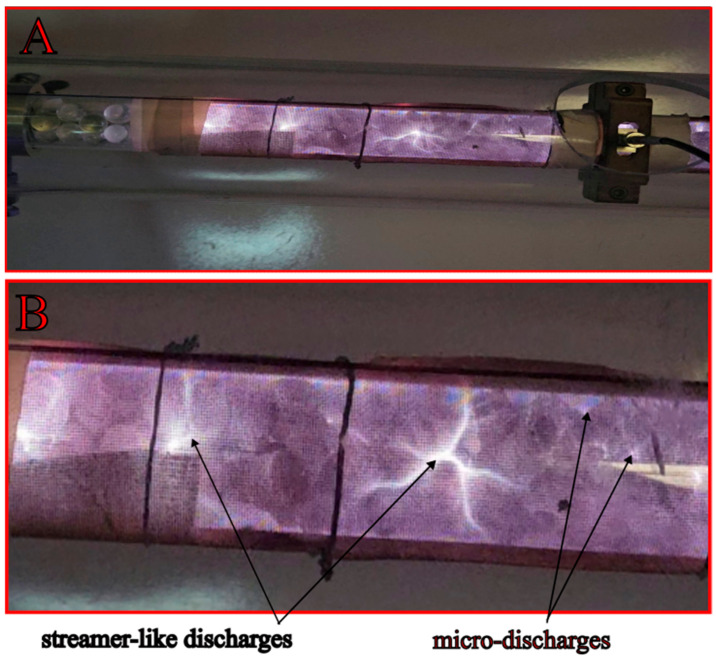
Exemplary imaging of plasma discharge in the DBD reactor packed with kaolin pellets (**A**); ((**B**) zoom).

**Figure 13:**
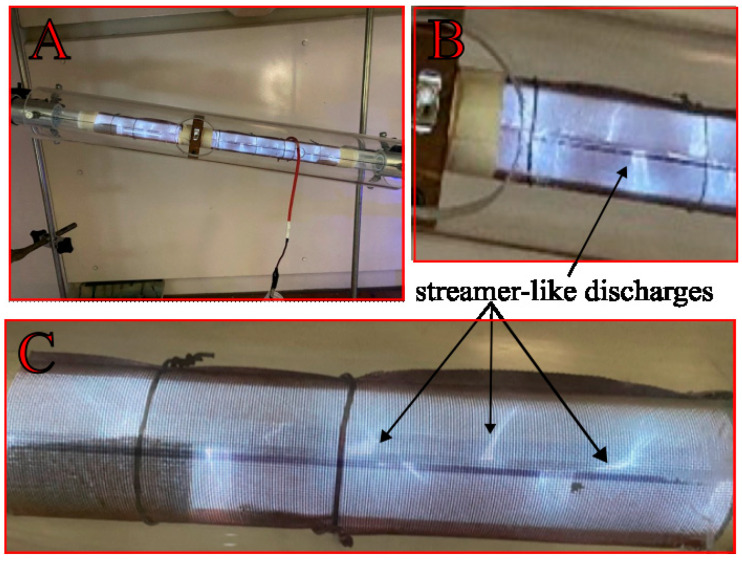
Exemplary imaging of plasma discharge in the empty DBD reactor (**A**); ((**B**,**C**) zoom).

To understand the interaction between the plasma and various packing materials, images of the operating reactor were captured under different conditions. The following Figure 11, Figure 12 and Figure 13 provide a qualitative comparison of the discharge propagation modes for the different geometries and materials investigated in this study.

The presence of a dielectric packing material fundamentally distinguishes a packed-bed dielectric barrier discharge (DBD-PB) reactor (Figure 11 and Figure 12) from its empty reactor counterpart (Figure 13). The introduction of a dielectric packing material into a DBD reactor fundamentally alters the nature of the plasma, yielding a more effective system for gas conversion. These findings are consistent with reports in the literature [6,26,28]. This is due to the creation of a strong, inhomogeneous electric field concentrated at the contact points of the pellets [41]. This localized field enhances electron energy and leads to a combined mode of filamentary and surface discharge, which is more stable and uniform than the filamentary discharge found in empty reactors. As can be seen in Figure 12, this phenomenon is particularly evident in the kaolin variant, which is characterized by a multitude of distinct, streamer-like discharges and clearly visible localized micro-discharges. This effect synergizes with the influence of flow turbulence on the plasma characteristics to contribute to satisfactory CO_2_ conversion results. The sharp edges of the kaolin pellets in the plasma reactor were found to promote higher local electric fields and more energetic electrons [42]. The gas discharge is initiated near the electrode and propagates along the dielectric surface as a creeping or sliding discharge [43]. This mechanism enables a better utilization of the reactor volume. On top of that, the occurrence of the observed discharge is independent of the packing bead size, given a sufficiently low dielectric constant (e.g., below 20 for glass). Consequently, a representative image of the plasma is presented for only one of the bead variants (glass 3 mm, Figure 11), and the discharge characteristics are visually similar for both corundum and zirconia. These phenomena and observations collectively contribute to a singular outcome. DBD-PB reactors offer superior conversion and energy efficiency compared to systems without packing. These combined effects promote CO_2_ decomposition.

### 3.6. Scaling Strategies for Industrial Packed-Bed DBD Reactors in CO_2_ Utilization

Scaling up PB-DBD plasma reactors for industrial CO_2_ utilization is challenging, as simple geometric enlargement degrades plasma uniformity and efficiency. Therefore, the most robust strategy is modularization (numbering up) to maintain optimal performance, although this still requires mitigating challenges specific to the packed bed, such as ensuring uniform gas flow to prevent channeling and managing pressure drop to conserve pumping energy. Crucially, the scaling strategy should favor the use of relatively inexpensive packing material alongside higher gas flow rates to leverage the improved reactor throughput (processing capacity) and minimize overall operational costs. Regardless of the architecture, successful scale-up is ultimately determined by maintaining the optimal specific energy input (SEI) necessary for consistent conversion.

### 3.7. Mathematical Modeling of a DBD Plasma Reactor

This paper attempts to develop a mathematical model describing CO_2_ conversion in a DBD reactor. A simple kinetic model was proposed. Based on measurement results for corundum, parameters for this kinetic model were selected. The kinetic model proposed in this work was validated only for a plasma reactor filled with corundum. Unfortunately, for the other materials used in the study, the nature of the discharges did not allow for the development of a simple kinetic model. Therefore, the focus was on only one material used for kinetic modeling. The selection of this model was preceded by a literature review of DBD reactors. Mathematical modeling of dielectric barrier discharge (DBD) reactors enables the description of plasma dynamics through systems of partial differential equations that account for charge transport, electric fields, and chemical reactions. A key component is solving Poisson’s equation for the electric field and continuity equations for electrons and ions. The models can be one-, two-, or three-dimensional and often employ either fluid or hybrid approaches (combining fluid and particle-based methods). Dielectric properties and microscopic phenomena, such as secondary electron emission and surface recombination, are also considered. Such simulations allow for the optimization of reactor geometry and power supply parameters regarding plasma stability and efficiency. One of the main challenges in describing the physicochemical parameters of DBD plasma is the difficulty of measuring local temperatures and species densities due to the strong spatial and temporal non-uniformity of the discharge. Dielectric barrier discharges typically consist of numerous micro-discharges, which makes averaged values of parameters—such as electron temperature or radical concentration—potentially unrepresentative of local reaction conditions.

Additionally, the lack of complete and accurate kinetic data for many chemical reactions occurring in atmospheric plasma hinders the development of reliable reaction models. Two main approaches to the modeling of dielectric barrier discharge (DBD) plasma reactors can be found in the literature. The first involves describing the balance of ions, electrons, and neutral atoms, along with Maxwell’s equations [44]. This approach is suitable for describing processes occurring in low-pressure gases. The second approach is based on dimensional analysis to develop correlations that allow for the calculation of key process parameters, such as the CO_2_ conversion rate [45], based on operational parameters characterizing the DBD plasma reactor. In DBD plasma reactors, the reactor packing plays a crucial role, as discharges occur on its surface, and low-temperature plasma is generated in its vicinity [46]. Therefore, a mathematical modeling approach based on chemical reactions occurring in a fixed-bed reactor was applied for the description of the DBD reactor. Since the total number of moles of reactants differs from the total number of moles of products, the mathematical model includes a molar flow balance for the gaseous species. Detailed modeling of fixed-bed reactor guidelines can be found in the work of Lewak [47].

The mathematical model is valid under the following assumptions:

- There is no internal diffusion within the pores of the packing material.
- The reaction rate is determined by the chemical reaction occurring in the plasma generation zone.
- The residence time in the reactor is on the order of several seconds, so molecular diffusion does not affect the mass transfer rate.
- Plasma is generated homogeneously throughout the reactor volume.
- The total pressure in the reactor has a constant value (*P* = const)

A single irreversible chemical reaction takes place in the reactor according to the stoichiometric equation:

(7) $${CO}_{2}\to CO+\frac{1}{2}O_{2},$$

The proposed reactor kinetic model was derived from a reactor used for dry methane reforming. Both processes are similar, although the mechanisms of carbon dioxide decomposition are entirely different. Both processes utilize a non-porous packing, thus eliminating internal diffusion resistance. Similarly, convective and diffusive transport of gaseous components can be ignored, as the residence times of the reactants in the reactor are very short. Therefore, convective mass transfer and molecular diffusion would be unable to influence concentration changes in the reaction space. The reaction mechanisms are similar. In a classical process, a catalyst and high temperature cause adsorption, reactions, and desorption of gaseous products. In the electrical discharge region, a low-temperature plasma is created, consisting of electron-depleted compounds that combine to form new products. The packing plays a different role than in a classical process. Nevertheless, its presence is crucial for plasma generation, thus acting as a quasi-catalyst. This makes the kinetic model applicable to DBD reactors. Validating it will provide important information about the process, even though it is a typical black box.

In low-temperature plasma, the energy of the electrons in the ionized gas has the primary influence on the process. Therefore, the model assumes that the energy input into the discharge is the primary factor. Therefore, the mathematical model does not consider the energy balance. The mathematical model has the following form.

(8) $$\frac{dN_{i}}{dx}=\nu_{i}\frac{1-\varepsilon }{\varepsilon }\cdot k_{DBD}\cdot F\cdot \rho_{solid}\cdot p_{CO2}^{n},$$

(9) $$y_{CO2}=\frac{N_{CO2}}{N_{CO2}+N_{CO}+N_{O2}+N_{Ar}},$$

(10) $$p_{CO2}=y_{CO2}\cdot P,$$

where

i = CO_2_, CO, O_2_, Ar and *ν*_CO2_ = −1; *ν*_CO_ = 1; *ν*_O2_ = −1/2; *ν*_Ar_ = 0;

*N*_i_—molar flow of reactants [mol s^−1^];

*ε*—porosity;

*ρ*_solid_—density of solid [kg m^−3^];

*F*—cross-sectional area of the reactor [m^2^];

*p*_CO2_—partial pressure [bar];

*k*_DBD_—constant reaction rate [mol s^−1^ bar^−n^];

*y*_CO2_—molar fraction of carbon dioxide;

*ν*_i_—stoichiometric coefficient of the i-th reactant.

**Figure 14:**
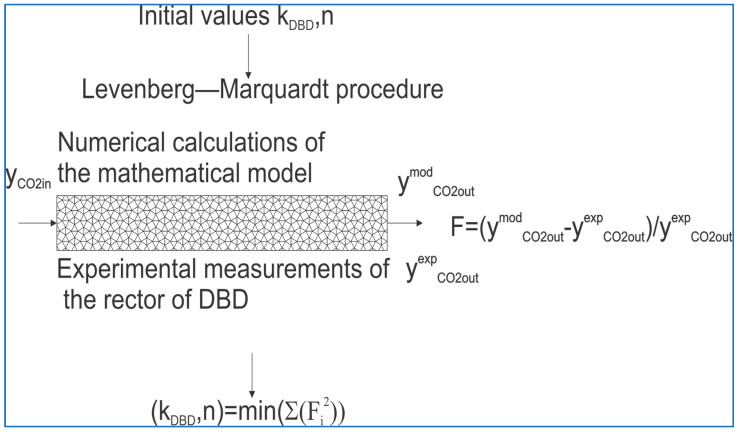
The schematic diagram of the optimization procedure.

The mechanism of carbon dioxide decomposition during barrier discharges is not fully understood, so the mathematical model was based on two parameters: *k*_DBD_ and n. The first parameter determines the rate constant of the CO_2_ decomposition reaction, and the second determines the exponential coefficient at the CO_2_ partial pressure. Both parameters characterize the production of carbon monoxide and oxygen during barrier discharges. The Levenberg–Marquardt algorithm, found in the lsqnonlin function available in the MATLAB R2024a package, was used to optimize the parameters in the kinetic model. The optimization procedure was based on relative error calculations using experimental measurements of carbon dioxide concentration at the reactor outlet and values obtained from numerical simulations performed in the MATLAB package. The ode15s procedure was used to integrate the system of ordinary differential equations (see the kinetic model). The procedure is presented in Figure 14. Using the lsqnonlin function, such parameters were sought in the kinetic model (see *k*_DBD_ and n) to achieve the minimum sum of squared relative errors.

**Figure 15:**
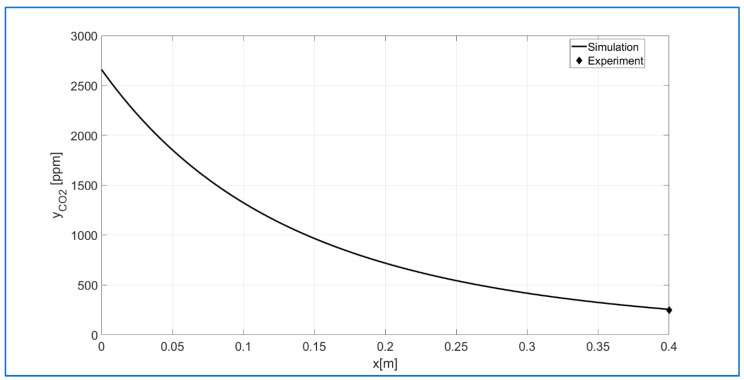
The numerical simulation for the best fit of experimental data to the kinetic model.

Figure 15 shows the results of a kinetic model simulation performed using the minimum sum of squared relative errors (see Figure 14). A point representing the measured CO_2_ concentration at the reactor outlet has been added to the Figure 15 plot. This fit is the best of the remaining data used for the nonlinear regression.

**Figure 16:**
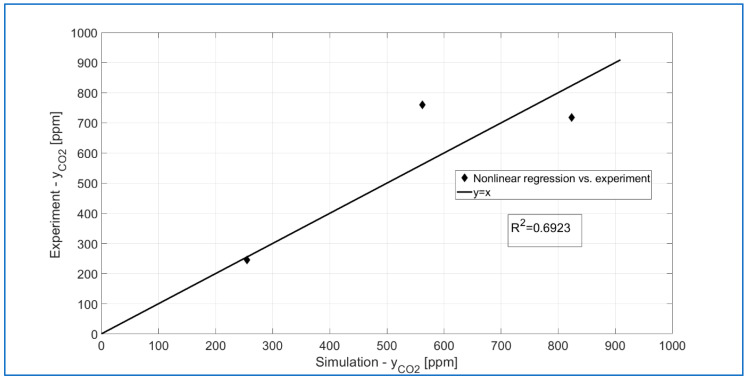
Results of fitting the kinetic model to the experimental data for corundum.

The results of the k_DBD_ and n parameter optimization procedure are presented in Figure 16. As can be seen, the CO_2_ concentration values at the outlet obtained from the numerical simulations describe the experimental results quite well. However, it must be admitted that these are preliminary results, and work on a better kinetic model will continue. As a result of the performed procedure, a local minimum of the F function was obtained (see Figure 14) for *k*_DBD_ = 0.0264 [mol s^−1^ bar^−n^] and n = 1.2004.

## 4. Conclusions

This study indicates that converting carbon dioxide using non-thermal plasma reactors has significant technological potential. However, plasma catalysis processes face a key challenge: balancing high conversion rates with high energy efficiency [48]. Increasing the gas flow rate reduces the specific energy input (SEI), which lowers conversion because less energy is available per mole of CO_2_. Conversely, higher flow rates improve energy efficiency. To address this issue, this work proposes combining a dielectric barrier discharge (DBD) reactor with a solid packing material that acts as a catalyst booster. The results demonstrate that selecting appropriate packing characteristics is crucial for achieving effective outcomes, as it helps to improve energy efficiency while maintaining high conversion levels. The two most effective materials for balancing high CO_2_ conversion and energy efficiency were identified as key options. Specifically, corundum achieved the highest conversion rate and relatively high energy efficiency, while 3 mm glass beads provided the highest energy efficiency with a moderately high conversion at an argon flow rate of 3 dm^3^/min. Since each material excelled in a different area, choosing the best option depended on the prioritization of a specific metric. To address this, we developed a new factor—mass-normalized conversion efficiency per energy unit—which considers the packing material’s mass and cost. Using this parameter, corundum showed a slight advantage over the glass beads. This minor difference supports selecting corundum as the top-performing packing material. However, if economic factors are primary, especially material cost, kaolin is the most advantageous, offering high conversion efficiency per energy unit with the lowest packing mass. This combination is important for the scalability and cost-effectiveness of plasma-based CO_2_ conversion. Overall, the choice of material depends on the specific goals of the process. In conclusion, our results highlight the importance of a comprehensive approach to plasma catalysis. Since plasma processes are often not fully understood by many researchers, it is essential to consider both physical and chemical effects together. A complete understanding of the process depends on recognizing the synergistic interaction between these effects, rather than treating them separately. Mathematical modeling of this phenomenon is limited by the challenge of accurately describing plasma discharges and physicochemical parameters. This constraint hinders the understanding of the chemical reactions, which are crucial for catalyst development. In this study, it is shown that a simple kinetic model, based on the partial pressure of the substrate, can be applied to complex systems such as dielectric barrier discharge (DBD) reactors. However, this model functions as a “black box,” providing only an approximation of experimental data without revealing the underlying mechanisms. Importantly, this study marks a significant methodological advance by representing the first instance of integrating plasma chemistry spectroscopic measurements (OES) into a DBD reactor to investigate the specific influence of the bed material on product formation. This approach provides an unprecedented, direct view of the plasma phase, offering critical, quantitative data on the discharge characteristics. This experimental strategy is pivotal for future mechanistic studies, allowing researchers to move beyond macroscopic performance metrics and establish fundamental correlations necessary for the rational design and true optimization of packed-bed plasma reactors.

## Figures and Tables

**Table 1:** The summary of the parameters of the packing beds utilized throughout the study.

	Glass 10 mm	Glass 5 mm	Glass 3 mm	Corundum	Zirconia	Kaolin
Pellet/bead diameter [m]	0.01	0.003	0.005	0.003	0.003	0.01
Pellet height [m]	NA	NA	NA	NA	NA	0.01
Number of pellets/beads [-]	176	1803	8121	8117	7796	211
Packing bed mass [kg]	0.257	0.303	0.333	0.276	0.421	0.122
Packing bed volume ×10^−4^ [m^3^]	0.922	1.180	1.148	1.147	1.102	1.657
Packing bed porosity [%]	32.593	41.736	40.605	40.585	38.980	58.611
Total surface area of pellets/beads [m^2^]	0.014	0.035	0.057	0.057	0.055	0.099

**Table 2:** The chemical composition of the glass support with different diameters.

		Concentration [wt.%]	
Compound	Glass 3 mm	Glass 5 mm	Glass 10 mm
Na	2.800	2.546	0.196
Mg	1.982	1.712	0.096
Al	1.290	1.126	0.482
Si	15.126	14.909	7.905
P	0.244	0.247	0.252
Cl	0.047	0.063	0.042
K	0.032	0.058	0.226
Ca	4.513	4.324	2.000
Fe	0.020	0.018	0.079
Zn	2.297	1.966	0.001
Zr	0.090	0.133	0.078
Mo	0.017	0.014	0.006
Sb	0.955	0.853	0.054
Ce	0.012	0.006	0.168
Ba	0.000	0.000	0.099

**Table 3:** The chemical composition of corundum, zirconia, and kaolin supports.

Corundum		Zirconia		Kaolin	
Compound	Conc. [wt.%]	Compound	Conc. [wt.%]	Compound	Conc. [wt.%]
Mg	0.123	Mg	0.299	Mg	0.307
Al	5.407	Al	25.903	Al	6.888
Si	16.304	Si	2.000	Si	12.623
P	0.254	P	0.230	P	0.272
Cl	0.004	K	0.031	K	1.020
K	2.211	Ca	0.965	Ca	0.924
Ti	0.063	Fe	0.053	Ti	0.140
Fe	0.191	Y	0.059	Fe	1.437
		Zr	7.986	Ni	0.050
		Ba	2.179	Zr	0.022
		Ce	0.020	Ba	0.055

**Table 4:** The specific energy input for all studied variants in the studied DBD-PB reactor.

	Mean Specific Energy Input ± SD [kJ/mol]						
Q Ar [dm^3^/min]	Glass 10 mm	Glass 5 mm	Glass 3 mm	Corundum	Zirconia	Kaolin	Empty Reactor
3	27.07 ±*1.72*	26.3 ±*1.46*	26.59 ±*1.45*	31.6 ±*3.95*	31.38 ±*6.08*	26.2 ±*0.78*	27.57 ±*2.03*
5	16.36 ±*1.03*	15.35 ±*0.7*	15.75 ±*0.81*	17.75 ±*3.08*	17.14 ±*3.1*	15.75 ±*0.5*	15.99 ±*0.28*
7	11.64 ±*0.66*	11.48 ±*0.77*	10.9 ±*0.49*	13.00 ±*2.19*	11.22 ±*0.25*	11.11 ±*0.44*	11.8 ±*0.3*

**Table 5:** The summary of performance metrics and trade-off evaluation for all studied variants in the studied DBD-PB reactor.

	**Absolute CO_2_ Conversion ± SD [%]**						
**Q Ar [dm^3^/min]**	**Glass** **10 mm**	**Glass** **5 mm**	**Glass** **3 mm**	**Corundum**	**Zirconia**	**Kaolin**	**Empty** **Reactor**
3	54.28 ±*2.73*	69.86 ±*9.77*	68.60 ±*5.67*	78.62 ±*1.61*	68.40 ±*1.19*	44.93 ±*5.32*	40.21 ±*0.11*
5	55.35 ±*2.11*	62.24 ±*1.76*	68.63 ±*0.68*	70.43 ±*8.78*	65.50 ±*2.29*	50.86 ±*1.41*	41.52 ±*0.32*
7	52.55 ±*3.11*	62.45 ±*0.35*	67.80 ±*1.83*	70.39 ±*7.8*	61.69 ±*3.34*	52.03 ±*1.29*	39.55 ±*2.99*
	**Energy Efficiency** ± SD [%]						
**Q Ar [dm^3^/min]**	**Glass** **10 mm**	**Glass** **5 mm**	**Glass** **3 mm**	**Corundum**	**Zirconia**	**Kaolin**	**Empty** **reactor**
3	1.52 ±*0.12*	2.01 ±*0.30*	1.95 ±*0.19*	1.88 ±*0.24*	2.04 ±*0.09*	1.30 ±*0.16*	1.10 ±*0.08*
5	2.49 ±*0.18*	2.99 ±*0.16*	3.21 ±*0.17*	2.93 ±*0.62*	3.15 ±*0.14*	2.38 ±*0.10*	1.92 ±*0.04*
7	3.29 ±*0.27*	3.97 ±*0.27*	4.54 ±*0.24*	3.95 ±*0.80*	4.21 ±*0.10*	3.42 ±*0.16*	2.44 ±*0.23*
	**Energy Cost** ± SD [kJ/mol CO_2_ reduced]						
**Q Ar [dm^3^/min]**	**Glass** **10 mm**	**Glass** **5 mm**	**Glass** **3 mm**	**Corundum**	**Zirconia**	**Kaolin**	**Empty** **Reactor**
3	18,659.94 ±*1508.86*	14,087.05 ±*2118.92*	14,501.49 ±*1435.58*	15,038.69 ±*1904.79*	13,856.18 ±*611.9*	21,822.36 ±*2665.76*	25,647.98 ±*1886.27*
5	11,344.57 ±*832.19*	9464.71 ±*510.21*	8808.43 ±*463.51*	9671.06 ±*2064.56*	8993.76 ±*405.04*	11,880.22 ±*502.56*	14,778.02 ±*295.58*
7	8594.93 ±*703.35*	7134.93 ±*482.62*	6236.16 ±*326.34*	7166.82 ±*1443.96*	6727.48 ±*158.93*	8284.27 ±*386.61*	11,579.3 ±*1112.25*
	**Mass-normalized Conversion Efficiency Factor Per Energy Unit** ± SD × 10^−7^ [mol CO_2_ reduced/(kJ·kg bed)]						
**Q Ar [dm^3^/min]**	**Glass** **10 mm**	**Glass** **5 mm**	**Glass** **3 mm**	**Corundum**	**Zirconia**	**Kaolin**	**Empty** **Reactor**
3	2.09 ±*0.17*	2.34 ±*0.35*	2.07 ±*0.21*	2.41 ±*0.31*	1.38 ±*0.27*	3.76 ±*0.46*	NA
5	3.43 ±*0.25*	3.49 ±*0.19*	3.41 ±*0.18*	3.75 ±*0.80*	2.37 ±*0.44*	6.9 ±*0.29*	NA
7	4.53 ±*0.37*	4.63 ±*0.31*	4.82 ±*0.25*	5.06 ±*1.02*	3.37 ±*0.20*	9.89 ±*0.46*	NA

**Table 6:** The selected argon transition lines for electron temperature determination.

λ [nm]	E_u_ [eV]	A_ul_ [1/s]	g_u_	Transition
420.0	14.49	9.67 × 10^5^	7	5p-4s
696.5	13.32	6.39 × 10^6^	3	4p-4s
706.0	13.3	3.80 × 10^6^	5	4p-4s
842.5	13.09	2.15 × 10^7^	5	4p-4s
852.0	13.8	1.37 × 10^7^	3	4p-4s

Where E_u_ is the energy of the upper level, A_ul_ is the transition probability, i.e., probability that one atom at the upper level will deexcite and emit a photon during one second, and g_u_ stands for the statistical weight of the upper level (g_u_ = 2J + 1, where J is the total angular momentum quantum number).

**Table 7:** Obtained values of electron temperature (in Kelvin). ND = No Data.

	Ar 3 dm^3^/min	Ar + CO_2_ 3 dm^3^/min	Ar + CO_2_ 5 dm^3^/min	Ar + CO_2_ 7 dm^3^/min
Empty reactor	ND	6306	6335	6312
Glass 3 mm	ND	ND	5631	5575
Glass 5 mm	5003	6162	5469	5509
Glass 10 mm	5424	5587	5504	5388
Corundum	4621	ND	5054	5287
Zirconia	3946	4440	4207	4368
Kaolin	4871	5724	5718	5765

**Table 8:** The selected spectral lines for detection of active species (subset of data given in [40]).

Species	λ [nm]	Upper Level	Lower Level	Detected
CO^+^	413.05	X^2^Σ	A^2^П	No
C_2_	436.08	X^3^П_u_	A^2^П_g_	Yes
CO^+^	471.01	X^2^Σ	A^2^П	Yes
C_2_	473.61	X^3^П_u_	A^3^П_g_	Yes
C_2_	516.40	X^3^П_u_	A^3^П_g_	Yes
C_2_	563.86	X^3^П_u_	A^3^П_g_	Yes
O_2_^+^	642.29	X^4^П_u_	b^4^Σ^−^_g_	No
O*	777.48	2s^2^2p^3^(^4^S^0^)3p	2s^2^2p^3^(^4^S^0^)3s	Yes

**Table 9:** The obtained intensity values for different plasma species measured at 5 dm^3^/min Ar+CO_2_ flow for different bed materials normalized to a 5 mm glass bed.

Species	λ [nm]	Line	Glass 10 mm	Glass 5 mm	Glass 3 mm	Corundum	Zirconia	Kaolin	Empty Reactor
CO^+^	413.05	CO+@413.05 nm	1.102	1.000	1.112	0.823	0.784	1.686	1.772
C_2_	436.08	C_2_@436.08 nm	1.123	1.000	1.212	1.122	0.743	1.279	1.325
CO^+^	471.01	CO+@471.01 nm	1.103	1.000	1.353	1.076	0.673	1.075	1.093
C_2_	473.61	C_2_@473.61 nm	1.101	1.000	1.359	1.106	0.804	1.039	1.037
C_2_	516.4	C_2_@516.4 nm	1.090	1.000	1.608	1.212	1.043	1.079	1.001
C_2_	563.86	C_2_@563.86 nm	1.109	1.000	1.601	1.136	1.089	1.443	1.220
O*	777.48	O*@777.48 nm	1.099	1.000	0.949	0.727	0.863	1.561	1.920

**Table 10:** Comparison of CO^+^ system line intensities normalized to the 777.48 nm O* line intensity for different bed materials (argon flow rate: 5 dm^3^/min).

λ [nm]	Glass 3 mm	Glass 5 mm	Glass 10 mm	Corundum	Zirconia	Kaolin	Empty Reactor
413.05	0.028	0.028	0.032	0.031	0.025	0.030	0.025
471.01	0.114	0.114	0.162	0.168	0.089	0.078	0.065
777.48 (O*)	1	1	1	1	1	1	1
MEDIAN	0.071	0.071	0.097	0.100	0.057	0.054	0.045
RANK	3	4	2	1	5	6	7

**Table 11:** Comparison of C_2_ system line intensities normalized to the 777.48 nm O* line intensity for different bed materials (argon flow rate: 5 dm^3^/min).

λ [nm]	Glass 3 mm	Glass 5 mm	Glass 10 mm	Corundum	Zirconia	Kaolin	Empty Reactor
436.08	0.070	0.069	0.088	0.106	0.059	0.056	0.048
473.61	0.123	0.123	0.175	0.186	0.114	0.082	0.066
516.4	0.265	0.267	0.453	0.446	0.323	0.185	0.139
563.86	0.088	0.087	0.146	0.136	0.109	0.080	0.055
777.48 (O*)	1	1	1	1	1	1	1
MEDIAN	0.105	0.105	0.161	0.161	0.112	0.081	0.061
RANK	4	5	2	1	3	6	7

## Data Availability

The original contributions presented in this study are included in the article. Further inquiries can be directed to the corresponding authors.
